# Intracranial Abscess and Proteus mirabilis: A Case Report and Literature Review

**DOI:** 10.7759/cureus.12326

**Published:** 2020-12-27

**Authors:** Rabia Muddassir, Asfandyar Khalil, Romil Singh, Shafaq Taj, Zoha Khalid

**Affiliations:** 1 Internal Medicine, Security Forces Hospital, Makkah, SAU; 2 Internal Medicine, Health Department Khyber Pahktunkhwa, Peshawar, PAK; 3 Internal Medicine, Metropolitan Hospital, Jaipur, IND; 4 Internal Medicine, Deccan College of Medical Sciences, Hyderabad, IND; 5 Internal Medicine, First Affiliated Hospital of Xi'an Jiaotong University, Xi'an, CHN

**Keywords:** intracranial abscess, brain abscess, proteus mirabilis

## Abstract

An intracranial abscess caused by *Proteus mirabilis *is rarely reported in adults. A 17-year-old girl presented with generalized tonic-clonic seizure, high-grade fever, headache, and vomiting with a history of slowly progressing apathy, clumsiness, and disorientation. She had meningeal signs and altered sensorium with a Glasgow Coma Scale of 10. The laboratory analysis revealed leukocytosis and elevated erythrocyte sedimentation rate. Brain computed tomography (CT) revealed a cystic lesion in the left temporal lobe with perilesional edema and a slight midline shift. She was commenced on empiric ceftriaxone, amikacin, and metronidazole. The non-foul smelling pus was drained through a craniotomy, and pus culture showed *P. mirabilis*. Culture sensitivity revealed extended-spectrum B-lactamase production, and she was commenced on intravenous carbapenem in addition to existing drugs. A repeat CT revealed a significant reduction in abscess size, and improvement in her condition was observed. On her recent follow-up visit, she was doing well.

## Introduction

Brain abscess is an intracranial infectious disease with a prevalence of 0.4-0.9 per 100,000 population and high disability and mortality rates [1]. Improvements in diagnosis and management of brain abscess and improved imaging modalities have reduced the mortality rates [2]. Brain abscesses occur due to intracranial invasion of an external infection via the hematogenous spread, direct spread, or any traumatic condition [3]. Among the microbial causes, streptococci are the most common resulting in brain abscess. *Proteus mirabilis* causes meningitis and brain abscess in the neonatal period and in immunocompromised patients. However, it is rarely reported in adults. Herein we present a case of brain abscess in a young girl due to *P. mirabilis.*

## Case presentation

A 17-year-old girl was brought by her parents with one episode of a generalized tonic-clonic seizure, intermittent high-grade fever, headache, and vomiting for the last seven days. She also had a history of slowly progressing apathy, clumsiness, and disorientation. She had no relevant past medical history and no history of weakness of limbs, chronic fever, or trauma. On initial evaluation, she had a temperature of 99°F, blood pressure of 120/85 mmHg, respiratory rate of 29 per minute, heart rate of 99 beats per minute, and oxygen saturation of 99% on room air. On physical examination, the patient had meningeal signs and altered sensorium with a Glasgow Coma Scale (GCS) of 10. Her power, coordination, and reflexes were intact, and her toes were down going. Examination of the respiratory system and cardiovascular system revealed a typical picture, and there were no significant ear, nose, and throat (ENT) findings. The initial laboratory analysis revealed leukocytosis and an elevated erythrocyte sedimentation rate (Table 1).

**Table 1:** Results of hematological examination. WBC, white blood cells; RBC, red blood cells; AST, aspartate aminotransferase; ALT, alanine aminotransferase; ESR, erythrocyte sedimentation rate.

Parameter	Lab value	Reference
WBC count, cells/mm^3^	16000	4000–10,000
RBC count, million cells/mm^3^	4.3	4.2 to 5.4
Hemoglobin, g/dL	14.2	14–17
Hematocrit, %	42.8	41–51
Platelet count, cells/mm^3^	179,000	150,000–350,000
Sodium, mmol/L	138	136–145
ESR, mm/hr	24	≤ 20
Potassium, mmol/L	3.9	3.5–5.0
Chloride, mmol/L	100	98–106
Urea nitrogen, mg/dL	12	8–20
Creatinine, mg/dL	0.9	0.7–1.2
Blood glucose, mg/dL	101	70–100 (fasting)
Total bilirubin, mg/dL	0.07	0.3–1.2
AST, IU/L	29	5–40
ALT, IU/L	39	7–56
Parameter	Lab value	Reference
WBC count, cells/mm^3^	16000	4000–10,000
RBC count, million cells/mm^3^	4.3	4.2 to 5.4
Hemoglobin, g/dL	14.2	14–17
Hematocrit, %	42.8	41–51
Platelet count, cells/mm^3^	179,000	150,000–350,000
Sodium, mmol/L	138	136–145
ESR, mm/hr	24	≤ 20
Potassium, mmol/L	3.9	3.5–5.0
Chloride, mmol/L	100	98–106
Urea nitrogen, mg/dL	12	8–20
Creatinine, mg/dL	0.9	0.7–1.2
Blood glucose, mg/dL	101	70–100 (fasting)
Total bilirubin, mg/dL	0.07	0.3–1.2
AST, IU/L	29	5–40
ALT, IU/L	39	7–56

**Figure 1:**
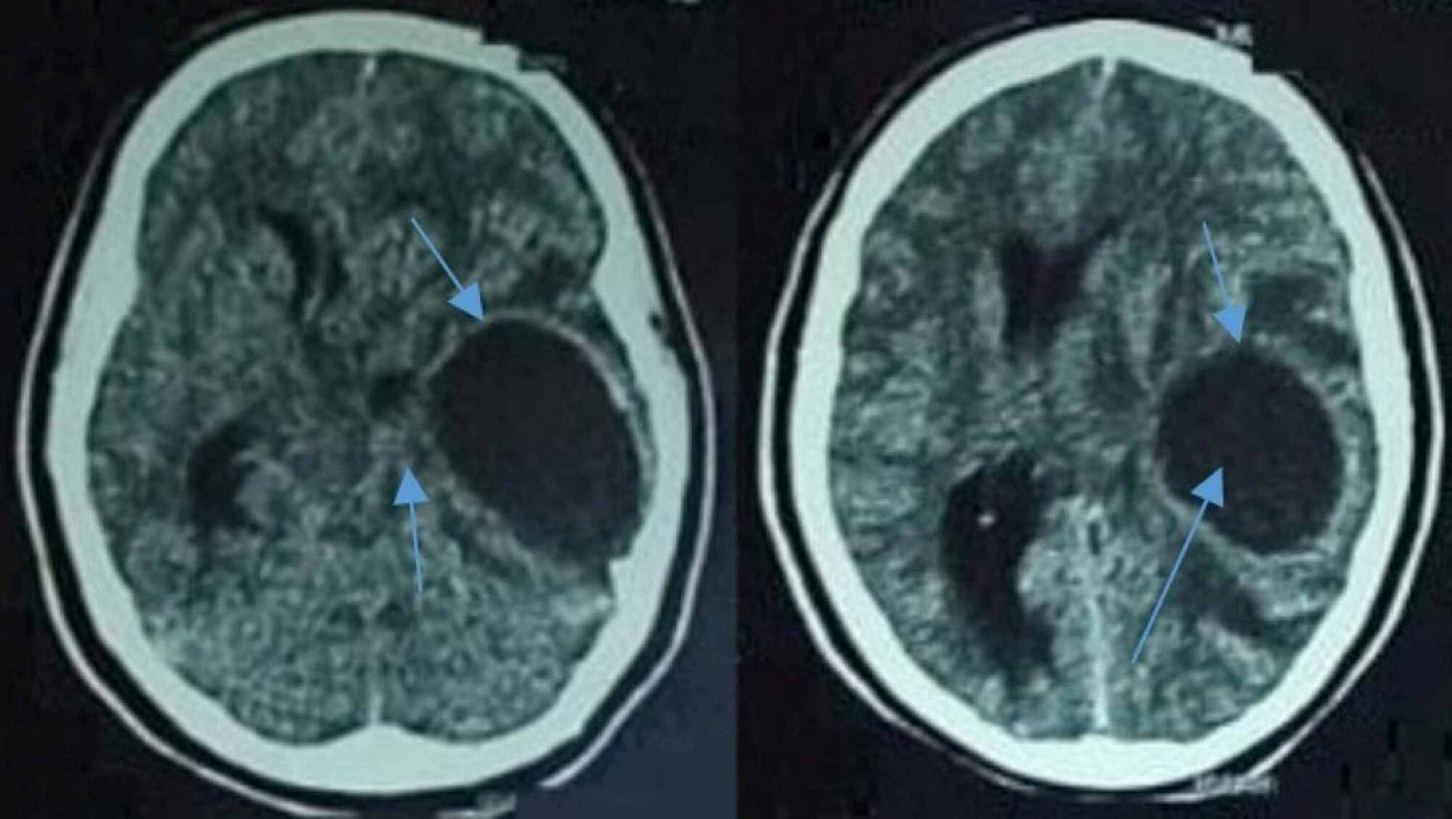
Axial images of the CT brain showing an enhancing cystic lesion with perilesional edema and midline shift (blue arrows). CT: Computed tomography

Her clinical picture was suggestive of the central lesion. Brain computed tomography (CT) was performed, which revealed a cystic lesion in the left temporal lobe with perilesional edema and a slight midline shift (Figure 1). A provisional diagnosis of temporal brain abscess was made. She was commenced on empiric ceftriaxone, amikacin, and metronidazole. A lumbar puncture was performed, which revealed pleocytosis (polymorphs 31 x 10^6^/L, lymphocytes 2.5 x 10^6^/L, glucose 1.6 mmol/L, and protein 0.45 g/L); however, the cerebrospinal fluid (CSF) culture showed no growth. 

**Figure 2:**
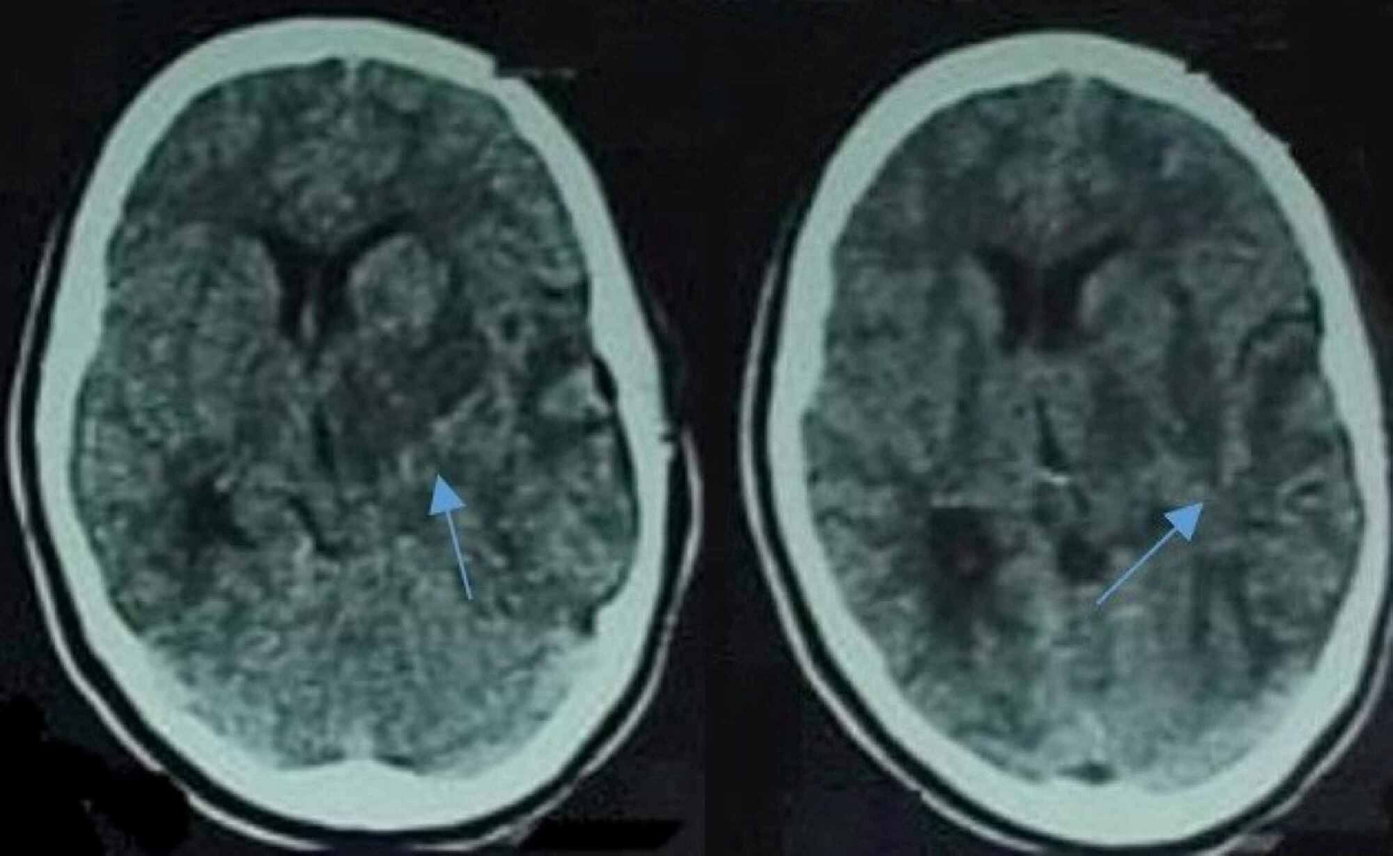
Axial images of CT brain showing significant reduction in abscess size after craniotomy and initiation of antibiotics (blue arrows). CT: Computed tomography

The patient went through emergency craniotomy, and non-foul smelling pus was drained and sent for investigation. The patient’s headache was alleviated with fluctuated fever. The culture showed *P. mirabilis* growth, and culture sensitivity revealed extended-spectrum B-lactamase production. She was commenced on intravenous carbapenem in addition to existing drugs. Cerebral edema was managed symptomatically. The patient’s condition improved, and she became conscious and well oriented. A repeat brain CT was performed, which revealed a significant reduction in abscess size (Figure 2). She was discharged after nine days of hospital stay and was referred to a hospital clinic for further observation and treatment. On her recent visit, the patient was doing well without any significant ENT and urinary pathology, and the abscess source was not known. She was advised to continue her antibiotics for further two weeks.

## Discussion

Brain abscess is a severe, life-threatening condition that most commonly occurs in young adults and middle-aged individuals, with a prevalence of 1.5-3.4 [4]. The streptococcus species are frequently associated with bacterial abscess. Bacterial brain abscess due to *P. mirabilis* is common in neonates. However, *P. mirabilis* is rarely seen in adults.

Brain abscess usually presents with headache, fever, and focal neurological signs and symptoms. Almost 5% of the patients develop all three symptoms, and an epileptic attack occurs in 28% of the patients. The abscess source is multifactorial and can be due to hematogenic, cardiogenic, post-traumatic, odontogenic, secondary to intracranial lesions, pulmonary infections, and urinary tract infections. However, in some cases, the patients have an insidious brain abscess source that cannot be identified; these cases are labeled as cryptogenic brain abscesses as in our case [3]. These patients have no apparent history of infection or physical signs, which result in difficulty in diagnosis.

Diagnosis of brain abscess usually depends on the patient's clinical picture and using imaging modalities such as CT, magnetic resonance imaging (MRI), magnetic resonance spectroscopy (MRS), and diffusion-weighted imaging (DWI). CT and MRS permit accurate identification and an enhanced picture of the intracranial abscess. However, DWI being more specific and sensitive is the most valuable method to diagnose brain abscess and differentiating it from brain malignancy. A brain abscess can be divided into meningoencephalitis, suppuration, and capsule stage based on imaging [2].

Brain abscess is managed with medication and surgery. Medicated treatment is suitable only during the early suppuration stage, for small abscess. It is inevitable to administer antimicrobial therapy as soon as possible [3]. Empiric medication may be commenced based on infection and lesion sites before the actual etiology and drug sensitivity are known. In polybacterial conditions, administration of cephalosporin combined with metronidazole can be given. Meropenem can be used as a substitute in the case of contraindications [2]. Periodic imaging should be performed to evaluate the therapeutic outcome. At this point, consolidation treatment should be given for at least two weeks. Surgical approaches for brain abscess comprise stereotactic resection and puncture aspiration based on lesion size, location, and presence of capsule of the lesion, severity of the disease, and patient's age. Effective management signs include improvement in clinical symptoms, mitigation or disappearance of the edema on imaging, and alleviation or fading of the lesion [3].

Our patient presented with meningeal signs and symptoms. She had an abscess with an intact capsule, and she was managed by medication and surgical intervention. Early recognition and confirmation by imaging and culture led to the successful management of the patient. It was a unique case of brain abscess due to* P. mirabilis *to the best of our knowledge.

## Conclusions

Cryptogenic brain abscess is rare in adults with normal immune responses. However, when a patient presents with a classic triad of headache, fever, and focal neurological deficits, a possibility of brain abscess must be ruled out based on history and physical examination. Although brain abscess due to *P. mirabilis* is rare in adults, it should be considered a differential diagnosis among the microbial causes. Early diagnosis and treatment are pivotal to prevent complications and disease mortality.
